# Imaging and clinical features of intra-abdominal injuries in children with suspected physical abuse

**DOI:** 10.1007/s00247-025-06335-x

**Published:** 2025-08-02

**Authors:** Tagrid M. Ruiz-Maldonado, M. Katherine Henry, Esther Ro, Shashank S. Prasad, Anna Kalathil Thomas

**Affiliations:** 1 Dept of Pediatrics, University of Utah, Salt Lake City, UT USA; 2 Center for Safe & Healthy Families, Primary Children’s Hospital, Salt Lake City, UT USA; 3 Safe Place: Center for Child Protection and Health, Clinical Futures, and Department of Radiology, Children’s Hospital of Philadelphia, Philadelphia, PA USA; 4 Department of Pediatrics, Perelman school of Medicine at the University of Pennsylvania, Philadelphia, PA USA; 5 Department of Medical Imaging, Ann & Robert H. Lurie Children’s Hospital of Chicago, Chicago, IL USA; 6 Department of Radiology, Northwestern University Feinberg School of Medicine, Chicago, IL USA; 7 Department of Radiology, The University of Texas Southwestern Medical Center, Dallas, TX USA; 8 Department of Radiology and Imaging, Children’s Hospital of Los Angeles, Los Angeles, CA USA; 9 Department of Radiology, University of Southern California, Children’s Hospital of Los Angeles 4650 Sunset Boulevard Mail Stop #4, Los Angeles, CA 90027 USA

**Keywords:** Diagnostic imaging, Abdomen, Injuries, Infant, Child, Physical abuse

## Abstract

The effective diagnosis and management of inflicted intra-abdominal injuries require a comprehensive, collaborative evaluation that includes a thorough understanding of the clinical presentation, laboratory findings, injury mechanisms, and potential clinical outcomes. This review presents the various aspects of inflicted intra-abdominal injuries, including the clinical presentation, mechanisms of injury, and the utility of available screening and imaging tools. The specific types of solid organ and hollow viscus injuries relevant to child physical abuse and their imaging appearance are also discussed. Radiological imaging provides critical information that can help treating providers contextualize the history obtained and the injuries identified, highlighting the value of close collaboration among radiologists, child abuse pediatricians, and other members of the clinical team when concerns for abuse arise.

## Introduction

More than 100,000 children are identified as victims of physical abuse in the USA annually [1]. This number likely under-represents the true burden of physical abuse as many victims are not recognized and remain unreported. Young children are disproportionately impacted, with infants having the highest risk of serious or fatal physical abuse [2]. Intra-abdominal injuries constitute a small but important subset of injuries from physical abuse.

Approximately 3% of children undergoing subspecialty child physical abuse evaluations are found to have intra-abdominal injuries [3, 4]. Compared to children with accidental intra-abdominal injuries, children with inflicted intra-abdominal injuries are typically younger, with infants and toddlers at highest risk [5–8]. An estimated quarter of hospitalizations for intra-abdominal injuries in infants and more than 1 in 10 hospitalizations for intra-abdominal injuries in children 1–2 years old are attributable to abuse [7]. While less common than other physical abuse injuries, identification of inflicted intra-abdominal injuries may be critical to informing the care and protection of the child.

Abdominal trauma may result in a broad spectrum of injuries that include injuries to solid organs, hollow viscus, mesentery, and vascular structures. In both inflicted and accidental trauma, solid organ injuries are more common than hollow viscus injuries [9, 10]. Injuries for both accidental and inflicted abdominal trauma in children are graded in accordance with the American Association for the Surgery of Trauma (AAST) Organ Injury Scale.

Most intra-abdominal injuries identified in the context of physical abuse are self-limiting and do not warrant intervention, except for certain bowel and pancreatic injuries [5, 9, 10]. Concurrent solid organ and hollow viscus injuries are more frequently reported in cases of physical abuse than in cases of accidental trauma [11]. Additionally, children with injuries concerning for physical abuse are reportedly more likely than children with accidental trauma to have injuries involving multiple abdominal sites, a higher injury severity score, and co-existing injuries such as fractures, oral injuries, bites, and burns as well as head and thoracic trauma (i.e., pulmonary contusions; rib and clavicle fractures) [5, 6, 11–13]. It is important to note that no intra-abdominal injury is pathognomonic for physical abuse. However, certain injuries are found to be overrepresented in cases of suspected abuse. Consideration of the type of abdominal injury in the context of the child’s age and development, reported mechanism of injury, and co-occurring injuries is necessary to inform the clinical assessment and determine the overall level of concern for abuse.

## Mechanisms of injury

Accidental abdominal trauma typically involves known mechanisms of injury. In contrast, cases of inflicted injuries often lack a clear history of trauma, making the mechanisms of injury less well-defined. Nonetheless, several proposed mechanisms have been described. A frequently reported mechanism of inflicted intra-abdominal injury is a direct blow to the abdomen (i.e., punching, kicking, slamming of the abdomen against a rigid surface). In addition to blunt trauma to underlying organs, direct blows may also result in compression of the intra-abdominal organs against the anterior spinal column or increased intraluminal pressure in blood vessels and hollow viscus organs [5, 10, 14]. Another published mechanism of injury involves sudden acceleration-deceleration resulting in shearing forces between fixed and mobile parts of the gastrointestinal tract, especially the bowel and mesentery. The resulting shearing forces commonly involve the duodenojejunal junction and ileocecal junctions [15]. Both direct compression injuries and shearing injuries can result in bowel perforations [5]. Additionally, mesenteric tears and contusions, which may accompany bowel injuries, can result in bowel ischemia and stricture formation [5, 14].

In discussing mechanisms of intra-abdominal injuries, falls warrant specific mention. Low-height falls and stair falls are common events prompting caregivers to seek medical attention and are often provided histories to account for inflicted intra-abdominal injuries [16, 17]. A retrospective study comparing pediatric patients with blunt abdominal trauma from abuse versus falls found that children under 5 years old who were victims of inflicted abdominal trauma had more pancreatic injuries, hollow viscus injuries, and/or severe head injury and had a higher injury severity score compared to patients who suffered a fall. Patients who suffered a fall were more likely to present with solid organ injuries [6]. Another retrospective study on pediatric abdominal injuries reported that small bowel injuries were more frequent with inflicted trauma in children 0–14 years old compared to accidental falls, while fall-related bowel injury was not identified in children under 5 years old [17]. Similarly, stair falls have not been found to commonly result in intra-abdominal injury, especially small intestine perforations [18]. Overall, low-height falls and stair falls are uncommon causes of intra-abdominal injury in childhood, and intra-abdominal injuries attributed to these mechanisms warrant careful consideration to determine whether the provided history plausibly accounts for the injury identified.

## Clinical presentation and evaluation

Children with inflicted intra-abdominal injuries can present across a broad clinical spectrum. At one end, they may have clinically occult abdominal trauma, where an intra-abdominal injury is present, but no abdominal signs or symptoms are evident. At the other end, injuries may be obvious, with clear abdominal symptoms such as severe pain or visible external trauma. Presentations can also be subtle and may include abdominal tenderness, distention, abnormal bowel sounds, as well as non-specific symptoms such as vomiting and irritability. Abdominal bruising, abdominal distention, and abdominal tenderness have been found to be strongly associated with intra-abdominal injury when studied in children < 5 years old undergoing evaluation for child physical abuse [3, 9]. However, the absence of abdominal bruising does not preclude serious abdominal injury and is reportedly absent in up to 80% of cases of children with inflicted intra-abdominal injuries [5]. Often, there is no reported history of abdominal trauma, and the physical exam may be confounded by co-existing injuries. In non-verbal children and infants, recognizing or interpreting non-specific abdominal findings is even more challenging, and some injuries, whether inflicted or accidental, may have delayed presentations. Thus, caution is needed when interpreting caregiver delay in seeking care as an indicator of abuse [11].

Due to the non-specific nature of common signs and symptoms, researchers have sought to develop screening methods to guide the radiological evaluation of suspected intra-abdominal trauma. The Pediatric Emergency Care Applied Research Network (PECARN) created a clinical decision rule for identifying children at low risk for medical intervention following blunt abdominal trauma [19]. However, this tool does not account for the potential impact a missed injury may have on the diagnosis of physical abuse, nor does it address children without a clear presenting history of trauma [20]. As a result, the PECARN clinical decision rule is not suited to guide imaging evaluation for suspected physical abuse, and guidelines for this specific patient population are still needed.

Laboratory tests have been studied as screening tools to guide radiological imaging for intra-abdominal injury. The American Academy of Pediatrics has acknowledged a role for hepatic transaminases as part of the evaluation for physical abuse, even in cases without acute abdominal symptoms [21]. Two multicenter studies proposed that alanine aminotransferase (ALT) and aspartate aminotransferase (AST) values above an 80 IU/L threshold may be useful indicators for abdominal CT imaging in physical abuse evaluations to screen for occult abdominal trauma [3, 4]. However, a recent single-center study in children undergoing evaluation for physical abuse concluded that abdominal CT imaging in children without signs or symptoms may be limited to cases where AST > 200 IU/L or ALT > 125 IU/L [22]. These studies reflect a lack of consensus on the transaminase threshold to prompt abdominal CT imaging in children with concerns for abuse and no known history, signs, or symptoms of abdominal injury. A recent multicenter study identified wide practice variability in the use of abdominal CT imaging when transaminase values exceeded 80 IU/L in children assessed by a Child Abuse Pediatrics team [23], supporting the lack of consensus. This study additionally found a 0.3% prevalence of occult abdominal trauma in children under 5 years of age undergoing evaluation for physical abuse, suggesting that universal screening with hepatic transaminases may not be necessary in this patient population when abdominal signs or symptoms are absent. Pancreatic enzymes and urinalysis have also been considered potential screening tools for intra-abdominal injuries in physical abuse evaluations, but subsequent studies have found these tests to have limited sensitivity [4, 24]. The current lack of uniform practice with laboratory screening supports the need for further development of evidence-based guidelines. Thorough history-taking and meticulous serial examinations remain essential to direct the clinical evaluation, including the decision to obtain laboratory testing and imaging studies.

## Current imaging modalities

### Computed tomography (CT)

In clinically stable patients with suspected inflicted intra-abdominal injury, a CT of the abdomen and pelvis with intravenous (IV) contrast in the routine portal venous phase is the study of choice to evaluate for intra-abdominal or intrapelvic injury. The addition of excretory-phase imaging is reserved for cases of suspected kidney or urinary collecting system injuries to detect post-traumatic urine leak [25, 26]. Excretory-phase imaging can be acquired using a single IV contrast administration with two separate CT acquisitions at the portal venous and excretory/delayed phases, or an alternate method with less radiation exposure is the split-bolus technique. In this technique, the amount of IV contrast is split into two separate IV contrast administration times for a single CT acquisition. The portal venous phase is based on the timing of the second contrast administration, and the excretory phase is based on the timing of the first contrast administration [27, 28]. This allows for simultaneous portal venous and excretory-phase imaging without additional radiation. Oral contrast administration is not routinely done prior to the CT with IV contrast, except in cases of suspected duodenal injuries [25].

### Ultrasound (US) and contrast-enhanced ultrasound (CEUS)

The American College of Radiology (ACR) appropriateness guidelines do not recommend US as an initial study to evaluate concerns for inflicted intra-abdominal injury [25]. While US is less sensitive than CT in the detection of intra-abdominal injury, it can still play a role in the trauma evaluation of children due to a few advantages. US is quicker to access, does not require sedation, and does not expose children to ionizing radiation. Focused assessment with sonography for trauma (FAST) is a limited screening ultrasound to detect hemoperitoneum as a sign of a serious intra-abdominal injury. In a hemodynamically unstable patient, FAST can guide next steps in management, such as emergent surgical intervention [29].

Conventional US of the abdomen is a more detailed exam than FAST with direct evaluation of the solid organs. In experienced hands, conventional US can be effective in detecting solid organ injury; however, minor parenchymal injuries can be missed [30–33]. This shortcoming may be acceptable in cases where concern for abuse is low and US is used to detect only clinically significant intra-abdominal injury that may require intervention. However, the detection of even relatively minor intra-abdominal injuries may be important in the setting of inflicted trauma given potential forensic implications.

In recent years, contrast-enhanced ultrasound (CEUS) has been shown to improve diagnostic accuracy of US for solid organ injury in the pediatric population, approaching that of CT [34, 35]. It is important to note that while CEUS may show promise as an alternative to CT, CEUS has been found to be limited in diagnosing certain types of intra-abdominal injuries and there is limited data on its performance in children undergoing evaluation for physical abuse. Specifically, CEUS falls short in detecting urine extravasation and in distinguishing urinomas from perinephric hematomas given that the administered contrast agent is not excreted by the kidneys [34–36]. CEUS is limited in evaluating the pancreas and retroperitoneal structures due to depth from transducer and bowel interposition. Lastly, CEUS is generally a targeted exam to evaluate the solid organs and has not proven useful for detecting bowel or mesenteric injury [34, 36, 37]. CEUS is currently not recommended as a first-line modality to detect intra-abdominal injuries in child physical abuse evaluations.

### Magnetic resonance imaging (MRI)

MRI is not routinely used in the evaluation of abdominal trauma, including inflicted intra-abdominal injuries, but can be useful to evaluate specific concerns such as pancreatic and biliary ductal injuries. MRI offers reliable soft tissue contrast and lacks ionizing radiation, making it useful for characterization of complex injuries. However, MRI of the abdomen and pelvis is inherently limited in assessing bowel pathology without an oral contrast agent. It has lengthy acquisition times, requires sedation in young children, and can be delayed due to the need to coordinate sedation and address issues raised by MRI safety screening. MRI of the brain and spine is frequently performed in the evaluation of suspected physical abuse. When evaluating spine MRIs, it is important to scrutinize the visualized portions of the adjacent posterior chest, abdomen, and pelvis for potential traumatic visceral findings, which would need further evaluation.

### Radiographs

Abdominal radiographs can reveal indirect signs of intra-abdominal injury, such as pneumoperitoneum, but have a low sensitivity and specificity for detecting solid organ or hollow viscus injury as well as intra-abdominal hemorrhage [38]. For this reason, abdominal radiographs are generally not recommended for the evaluation of intra-abdominal injury in cases of suspected abuse. In physical abuse evaluations, particularly in young children, X-rays are often obtained, whether in the form of a skeletal survey or dedicated views of suspected injury sites [25]. Should these radiographs elicit concern for intra-abdominal injury, clinical correlation is warranted, and appropriate abdominal imaging should be considered.

## Specific intra-abdominal organ injuries

### Liver

**Fig. 1:**
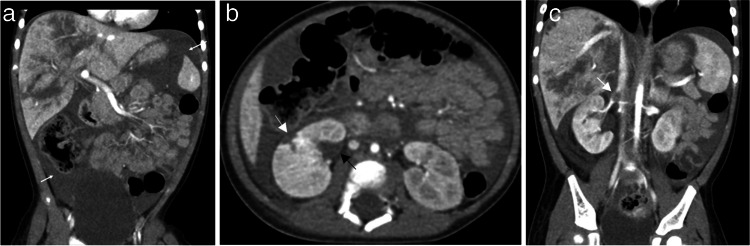
An 18-month-old male presented with lethargy and markedly elevated liver enzymes. Caregiver confessed to multiple blows to the epigastrium. **a** Coronal image of a post-contrast CT abdomen and pelvis in portal venous phase demonstrates extensive liver lacerations and a large volume complex peritoneal fluid (*white arrows*) with a higher density than the bladder. **b** Axial CT image from the same study shows two separate renal lacerations in the upper pole of the right kidney (*white and black arrows*). **c** Coronal image from the same study shows a post-traumatic right renal vein thrombus (*white arrow*)

**Fig. 2:**
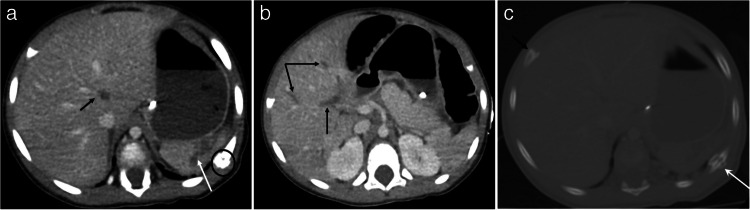
2-year-old male found unresponsive with chest wall bruising and elevated liver enzymes. Findings elicited concern for physical abuse. CT of the abdomen and pelvis in portal venous phase was obtained. **a** Axial image shows splenic (*white arrow*) and liver (*black arrow*) lacerations each consistent with grade II injuries and a healing rib fracture (*black open circle*). **b** Axial image of the same study at a slightly more caudal level shows multiple branching liver lacerations (*black arrows*) consistent with a grade II liver injury. **c** Axial CT image in bone algorithm at the level of the splenic laceration better delineates the healing left rib fracture (*white arrow*) and an anterior right rib fracture (*black arrow*)

**Fig. 3:**
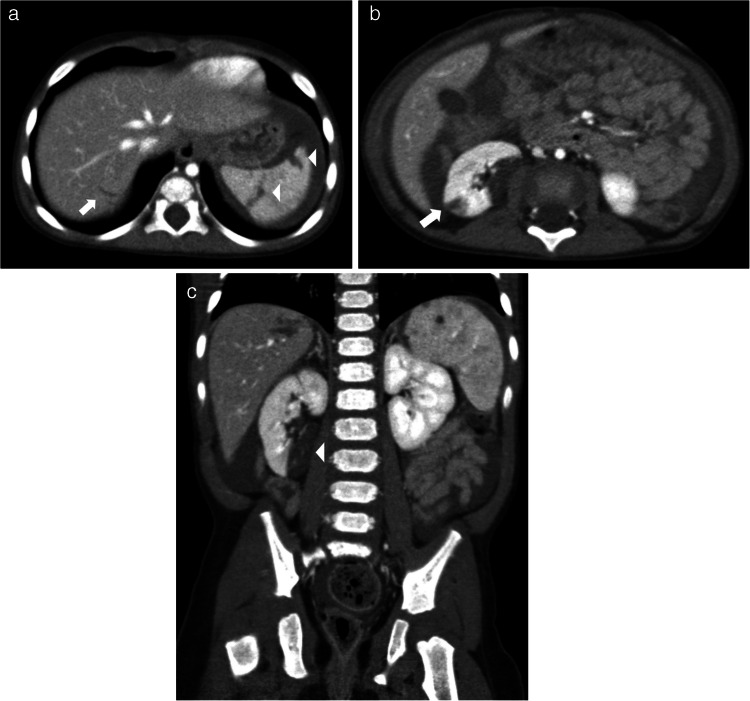
A 17-month-old male presented with lethargy, vomiting, extensive facial and neck bruising, and subconjunctival hemorrhages, concerning for physical abuse. **a** CT abdomen pelvis in portal venous phase shows grade II splenic (*white arrowheads*) and liver (*white arrow*) lacerations. **b** Axial image demonstrates a right renal laceration (*white arrow*). **c** Coronal image of the same study shows segmental devascularization injury of the renal lower pole (*white arrowhead*). Ascites as well as previously noted hepatic and splenic lacerations are also visible in the image (*unmarked*)

The liver is the most common solid organ injured in cases of inflicted intra-abdominal injury, likely attributed to its size and anatomic location [9, 11]. Nationally, liver injuries account for 64% of all abdominal visceral injuries in hospitalized children with suspected physical abuse, with liver lacerations representing the most commonly identified injury [7, 11]. Single-institution publications report that liver injuries represent 33% to 42.9% of intra-abdominal injuries in children diagnosed with physical abuse [9, 10, 13]. Liver injuries include contusions, lacerations, and subcapsular hematomas; rarely, diffuse organ disruption may result in cases of severe injury, and injuries may be associated with vascular and biliary complications [5, 14] (see Figs. 1, 2, and 3). On CT, contusions present as ill-defined hypodense areas, and lacerations appear as irregular, hypodense defects that traverse the parenchyma to the capsular surface [39]. Subcapsular hematomas are collections of blood deep to the capsule and appear as lens-shaped, frequently hypodense compared to the liver parenchyma, which can often be compressed by this collection. Sometimes subcapsular collections have scattered hyperdense areas, which are more likely to represent clotted blood than active extravasation, but delayed phase imaging can be helpful to look for contrast pooling to identify active bleeding [40]. The AAST grading severity increases with the depth of lacerations and the size of hematoma [41]. Congenital fissures or clefts and diaphragmatic indentations or slips may mimic hepatic injuries on CT imaging [42]. While complications can occur with more severe injuries to the liver, including arterial pseudoaneurysm, delayed hemorrhage, and biliary leak, most inflicted liver injuries are managed conservatively [5, 9, 10, 40, 43].

### Spleen

In cases of inflicted intra-abdominal injuries, splenic injuries are less common than liver injuries. Nationally, splenic injuries reportedly account for 9% of all injuries in hospitalized children with suspected physical abuse [7]. Single-institution publications report that splenic injuries represent 13.4% to 21% of intra-abdominal injuries in children diagnosed with physical abuse [9, 10, 13].

As with liver injuries, splenic injuries also include contusions, lacerations, and subcapsular hematomas, where the AAST grading severity increases with the depth of lacerations and the size of hematoma [41] (see Figs. 2 and 3). Higher grades are assigned to splenic injuries when the injury extends into the hilum due to a higher risk of hemorrhage. A splenic laceration involving two surfaces is referred to as a “fractured” spleen, and when multiple splenic parenchymal fractures are present, the spleen is termed “shattered” [40]. Congenital fissures or clefts and diaphragmatic indentations or slips may also mimic splenic injuries on CT imaging, as seen with liver injuries [42]. Most inflicted spleen injuries are managed conservatively [5, 9, 10, 42, 43]. In rare cases where splenic injuries result in hemodynamic instability and require intervention, vascular embolization is being increasingly utilized over intra-abdominal surgery [44].

### Pancreas

Nationally, pancreatic injuries account for 7.3% of all abdominal injuries in hospitalized children with suspected physical abuse [7]. Single-institution publications report that pancreatic injuries represent 9.8% to 16.7% of intra-abdominal injuries among children with physical abuse [9, 10, 13]. Pancreatic injuries are overrepresented in cases of inflicted intra-abdominal injury [6, 7, 10]. Pancreatic injuries related to physical abuse can be seen with hollow viscus injuries and may have a delayed presentation with pancreatic pseudocysts.

**Fig. 4:**
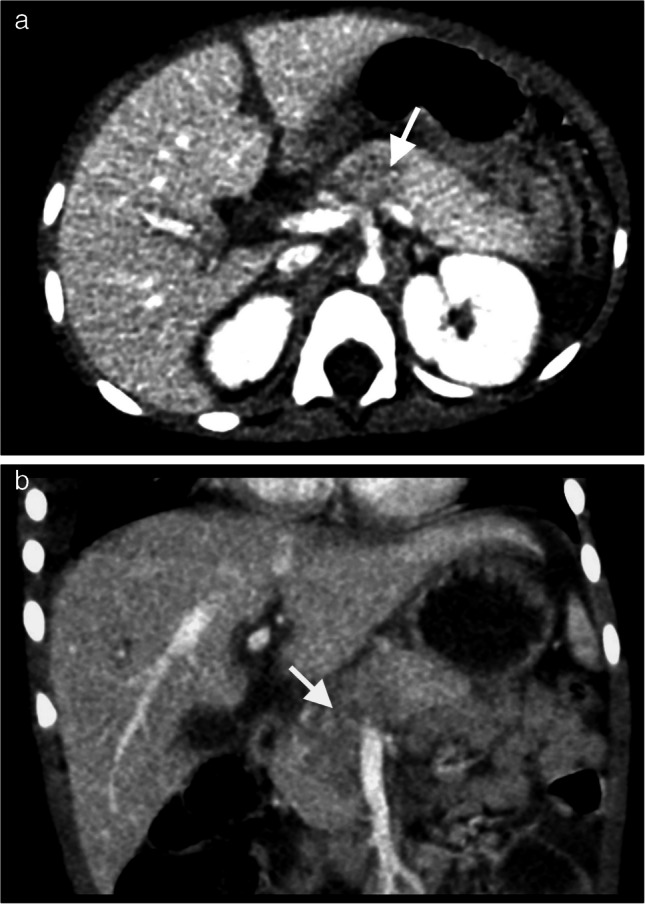
A 3-year-old female with multiple abdominal injuries from physical abuse including grade II left hepatic lobe lacerations, right adrenal hematoma, and a pancreatic contusion with elevated serum lipase. CT of the abdomen and pelvis with IV contrast was obtained in the portal venous phase. **a** Axial image at the level of the pancreas shows focal pancreatic body contusion (*white arrow*). **b** Coronal image shows the pancreatic body contusion (*white arrow*)

**Fig. 5:**
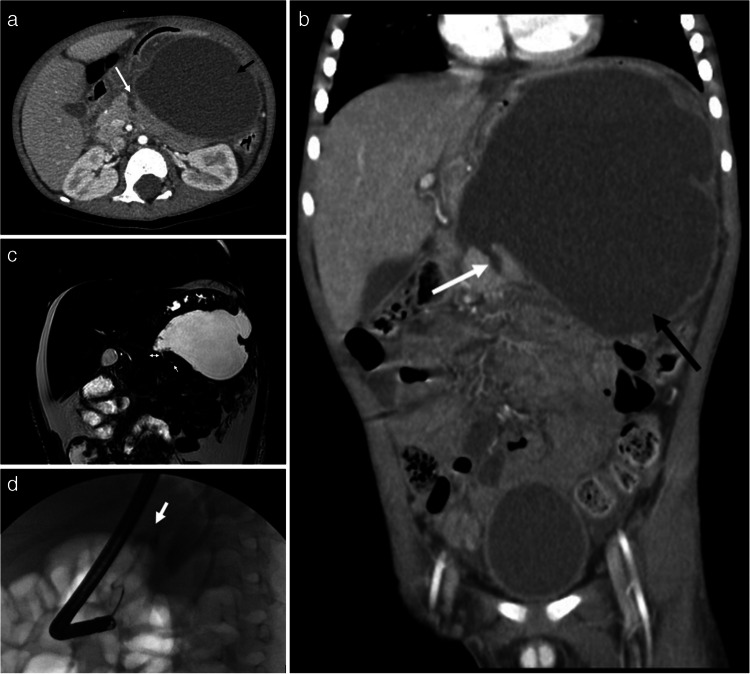
A 3-year-old male with a history of developmental delay presented with a new onset of repeated vomiting after feeds and on further evaluation was found to be in an abusive home environment. CT of the abdomen and pelvis with IV contrast in portal venous phase was obtained. **a** Axial image shows a deep laceration through the pancreas (*white arrow*) and a pancreatic pseudocyst anterior to the pancreatic tail (*black arrow*) and posterior to the stomach, which is partially compressed and anteriorly displaced by the pancreatic pseudocyst. **b** Coronal image shows a deep laceration through the pancreas (*white arrow*) which appears to communicate with a pancreatic pseudocyst (*black arrow*). **c** Coronal 3D thin slice MRCP image shows partial visualization of the main pancreatic duct in the pancreatic tail (*white arrow*) and a pancreatic laceration which communicates with the pancreatic pseudocyst near the main pancreatic duct course (*white double arrow*) concerning for a ductal tear. **d** Coned image from an ERCP with contrast injection into the main pancreatic duct shows a focal amorphous region of contrast extravasation (*black arrow*) in the region of the superiorly positioned pseudocyst confirming a tear of the main pancreatic duct which communicates with the pseudocyst

On CT, pancreatic injuries can present as focal gland enlargement, ill-defined, hypodense parenchymal contusions, lacerations, full-thickness gland transections, and pancreatic pseudocysts [5, 12, 14] (see Figs. 4 and 5). It should be noted that the diagnostic accuracy of CT for pancreatic injury in pediatric patients is limited. This is, in part, due to the relative paucity of fat in children, which limits peripancreatic fat stranding as a marker of injury, and due to some post-traumatic findings, such as fluid in the lesser sac, peripancreatic edema, and peripancreatic fluid, taking up to 24 h to manifest [14, 45, 46].

The integrity of the main pancreatic duct is the most important factor in determining whether emergent intervention is warranted for management of a pancreatic injury. Imaging findings of complete pancreatic transection, large lacerations exceeding 50% of the gland thickness, or a large amount of peripancreatic fluid on CT raise concerns about the integrity of the main pancreatic duct. These findings warrant further assessment with an MRCP [47, 48]. CT has limited sensitivity for detecting main pancreatic duct injury even when pancreatic parenchymal lacerations exceed 50% of the thickness of the gland [45, 46]. There is a paucity of information regarding the sensitivity and specificity of CT for identifying pancreatic duct injuries in children, but in adults, the reported CT detection rate for pancreatic duct injury is low, approximating 43% [45, 46]. Either MRCP or ERCP can be used to evaluate main pancreatic duct injury [45, 46]. In clinical practice, MRCP is typically preferred in the initial workup of pediatric patients with suspected main pancreatic duct injuries, as ERCP requires sedation and endoscopy, and thus, is reserved for complex cases needing further clarification.

Typically, distal pancreatectomies or, less commonly, pancreatic duct stenting are the interventions performed in children with main pancreatic duct injuries. However, there is a growing body of literature advocating for non-operative management in this setting [45, 47].

### Kidneys

Nationally, renal injuries account for 19.2% of all abdominal injuries in hospitalized children with suspected physical abuse [7]. Single-institution publications report that renal injuries represent 7.3% to 20% of intra-abdominal injuries among children diagnosed with physical abuse [9, 10, 13]. Renal injuries include renal contusions and lacerations, perinephric hematomas, and renal vascular injury (see Figs. 1 and 3).

CT of the abdomen and pelvis with IV contrast in both portal venous and excretory phases is the recommended modality to diagnose injury to the renal parenchyma and collecting system [25]. The routine use of excretory phase is not recommended for trauma workup and should only be used in select cases where renal or genitourinary tract injury is suspected. Hematuria is not a reliable predictor for renal injury, but the presence of gross hematuria or microscopic hematuria with > 50 RBC/hpf, with associated flank ecchymosis, rib fractures, or drop in hematocrit raises suspicion for renal or urinary tract injury [49]. CT can reliably depict renal contusions, depth of renal lacerations, perinephric hemorrhage, and major renal vascular injury or parenchymal devascularization. Renal contusions represent small intrarenal hematomas and appear as ill-defined, focal hypodense areas on CT. Excretory phase CT imaging depicts injury to the collecting system in the form of urinary contrast extravasation. In addition, the excretory phase can be useful to differentiate perinephric hematomas and urinomas and to diagnose urine leak in the setting of significant unexplained free intraperitoneal free fluid [49].

### Bladder

**Fig. 6:**
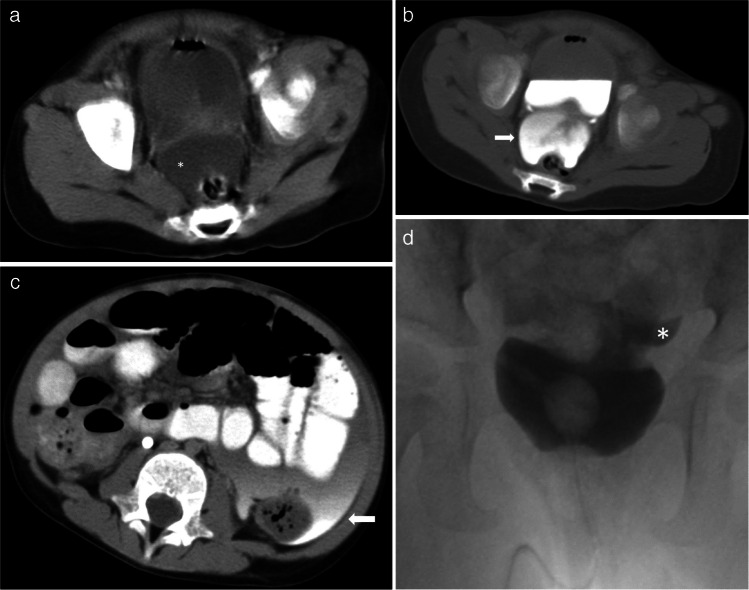
A 2-year-old female presented with abdominal bruising, abdominal pain, and acute renal failure without a history of trauma. Findings elicited concern for physical abuse. **a** Axial image at the level of the bladder in routine portal venous phase shows low-density fluid in the cul-de-sac (*white asterisk*). Air in the bladder from a recent Foley catheter placement is also visible (*unmarked*). **b** Axial CT image at the level of the bladder in excretory phase demonstrates contrast opacifying the cul-de-sac fluid consistent with urine leak (*white arrow*). **c** Axial CT image of the same study in excretory phase at a more cephalad location from the bladder shows contrast extravasation into peritoneum (*white arrow*). **d** Cystogram performed on the same patient through a Foley catheter shows contrast leakage from the bladder revealing an intraperitoneal bladder rupture (*white asterisk*)

Bladder injury is an uncommon occurrence with abdominal trauma. There are several case reports of bladder rupture from inflicted trauma [50, 51]. The mechanism of injury is hypothesized to be blunt trauma on a full urinary bladder causing intraperitoneal bladder rupture, typically at the dome of the bladder, which is the weakest part covered by the peritoneum. Spillage of urine into the peritoneal cavity can lead to peritonitis. If the bladder rupture has occurred more than 24 h prior to presentation, patients can present with electrolyte imbalance and azotemia from peritoneal absorption of urine, mimicking renal failure [50, 51]. This is particularly relevant in cases where there may be a delay in seeking medical care. Bladder rupture can present as non-specific intraperitoneal free fluid on US and portal venous phase CT without other intra-abdominal organ injury. An excretory-phase CT can indicate a bladder rupture with the presence of free extravasated contrast in the pelvis (see Fig. 6). Though bladder rupture may be identified on excretory phase CT imaging, a cystogram is the preferred approach to allow for adequate bladder distension resulting in a more thorough evaluation for intraperitoneal or extra-peritoneal bladder rupture. Intraperitoneal bladder rupture generally requires operative repair, but there are reports of successful conservative management with bladder catheterization and peritoneal drainage [50].

### Adrenal glands

**Fig. 7:**
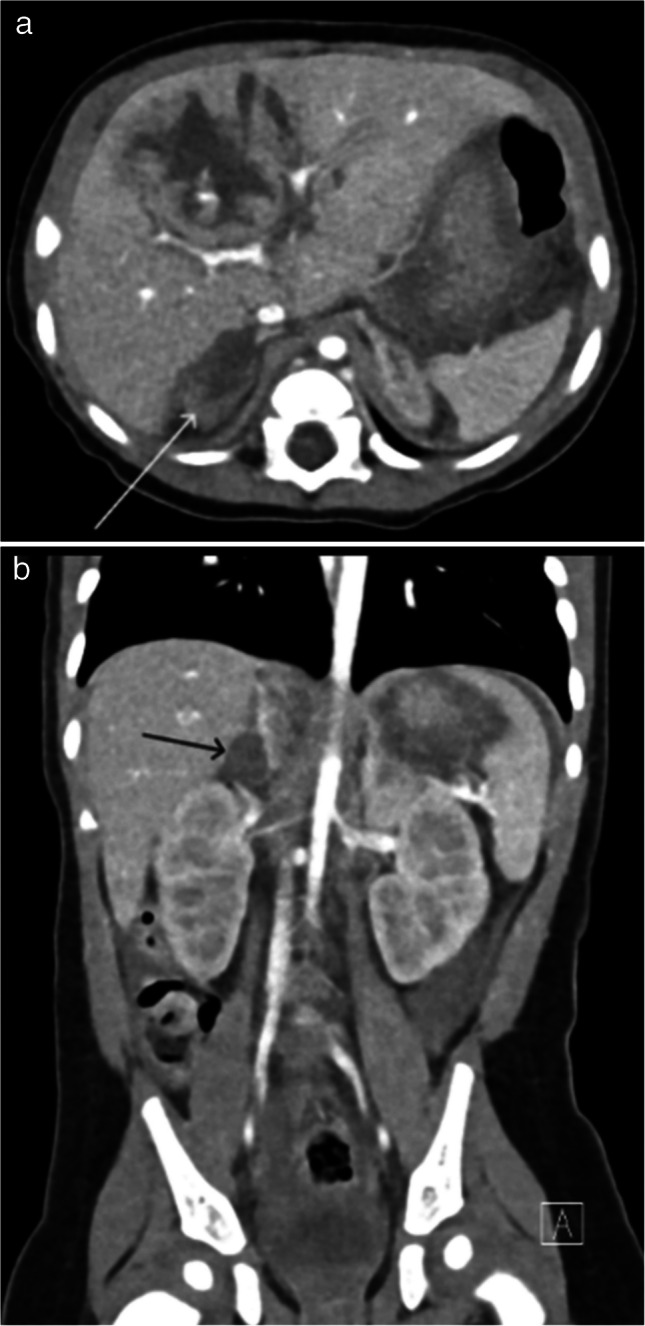
A 5-month-old male who was brought to the emergency department by his aunt for sleepiness and bruising over face, neck, chest, abdomen, and thighs. Further workup revealed bilateral rib fractures of different ages and elevated liver enzymes. **a** Axial contrast-enhanced CT scan of the abdomen in portal venous phase shows an oval, hypodense right adrenal hematoma (*white arrow*). The liver shows an area of branching lacerations, consistent with co-existing hepatic injury. **b** Coronal contrast-enhanced CT image of the same study shows the oval hypodense right adrenal hematoma (*black arrow*) which splays the adrenal limbs

Adrenal injuries are rarely reported in isolation in the context of physical abuse. When present, they often occur with multiple co-existing intra-abdominal and extra-abdominal injuries [9, 52]. Single-institution publications report that adrenal injuries represent 4.9% to 16.6% of intra-abdominal injuries among children with diagnosed physical abuse [9, 10, 13]. Adrenal hematomas are the most common type of injury and tend to involve the right side more frequently [53, 54] (see Fig. 7). Adrenal lacerations have been reported, albeit rarely, in the setting of fatal physical abuse and are also more likely to be right-sided [52]. The proximity of the right adrenal gland to the spine makes it more susceptible to compression during trauma, and its injury is often accompanied by ipsilateral visceral injuries, particularly to the liver and right kidney [55]. On CT and US, adrenal hematomas typically appear as an oval, mass-like structure often splaying the limbs of the adrenal gland, while MRI performed in the subacute phase reveals the presence of subacute blood products [54]. Over time, the hematoma undergoes characteristic changes, usually resolving completely, though residual calcifications may occasionally persist [56].

### Duodenum, proximal jejunum, and other hollow viscus organs

Nationally, hollow viscus injuries account for 12% of all abdominal injuries in hospitalized children with suspected physical abuse [7]. Although less frequent than solid organ injuries, hollow viscus injuries are overrepresented in cases of physical abuse and are more likely to require operative intervention [8, 10, 11]. Multiple sources report the small bowel, specifically the duodenum and proximal jejunum, as the most frequently injured hollow viscus organs in physical abuse [5, 6, 12, 17, 57]. Although less commonly encountered than small bowel injuries, the stomach and colon can also become injured from physical abuse [5, 58]. Acute hollow viscus injuries can range from mural hematomas to perforations, and long-term manifestations include bowel ischemia with associated luminal stenosis and strictures due to injuries of associated mesenteric vessels [5, 14].

In young children, duodenal injuries are strongly associated with abuse and have been the first recognizable sign of maltreatment in some reports [5, 12, 57, 59]. A retrospective study of patients 0–5 years old admitted with duodenal injuries at six different level 1 pediatric trauma centers over a 10-year period reported that physical abuse was responsible for all cases of duodenal injury in patients under 2 years old [59]. Additionally, a systematic review found that in children under 4 years old, duodenal injuries, particularly involving the third and fourth parts of the duodenum, were reported in cases of inflicted injury while no duodenal injuries were reported in the context of accidental trauma [5].

**Fig. 8:**
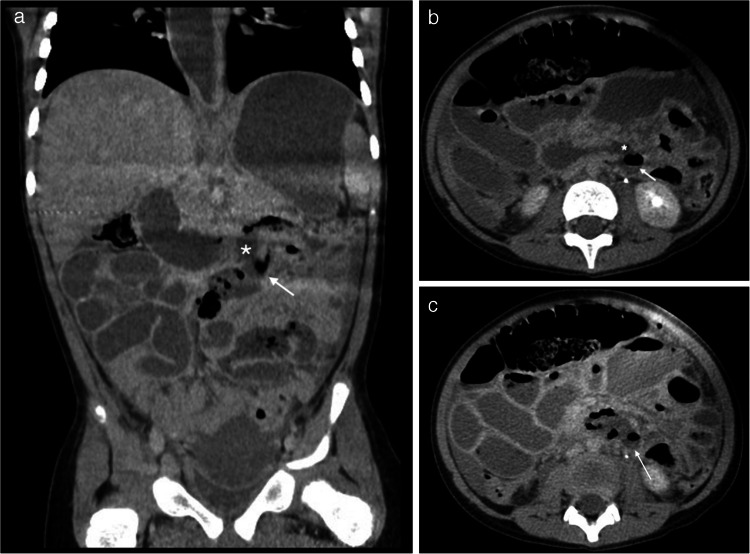
A 3-year-old male presented with a rigid abdomen after a reported low-height fall. CT of the abdomen and pelvis with only IV contrast using split-bolus technique revealed duodenal perforation that was subsequently confirmed at surgery. Findings elicited concern for physical abuse.** a** Coronal image shows a focal disruption of the inferior wall of the distal duodenum (*white asterisk*) which communicates with a collection containing extraluminal air and fluid (*white arrow*). **b** Axial image shows a focal disruption of the posterior wall of the distal duodenum (*white asterisk*) which communicates with the retroperitoneal collection containing fluid and extraluminal air (*white arrow*). **c** Axial image at a more caudal location better delineates a sizable retroperitoneal collection containing fluid and extraluminal air (*white arrow*) exerting mass effect on the adjacent bowel which is displaced anteriorly

**Fig. 9:**
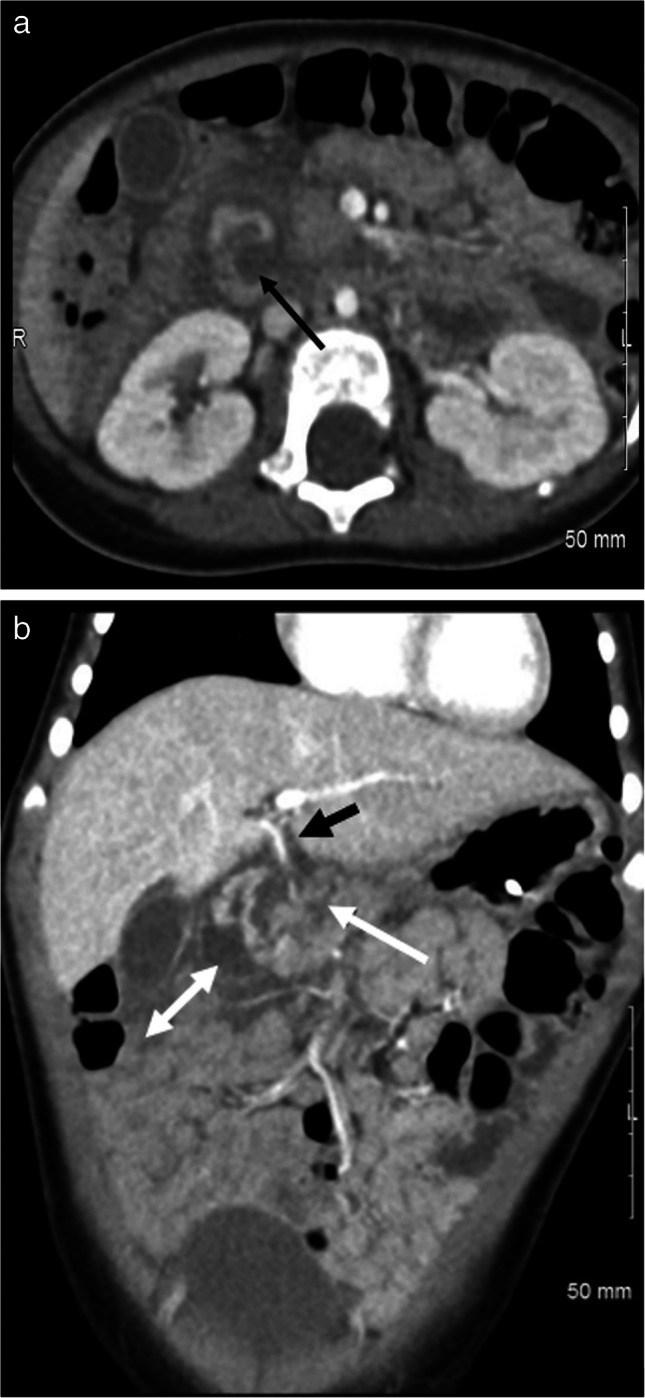
A 13-month-old male with no reported trauma history who presented with bilious emesis and underwent a CT of the abdomen and pelvis with IV contrast and no oral contrast in portal venous phase. Findings elicited concern for physical abuse. **a** Axial image shows a focal intramural lesion compatible with a duodenal hematoma (*black arrow*) in the medial wall of the second portion of the duodenum, near the ampulla of Vater. **b** Coronal image of the same study shows a dilated common duct (*black arrow*) and pancreatic duct (*white arrow*) due to obstruction of the ampulla of Vater by the duodenal hematoma (*double-headed white arrow*)

**Fig. 10:**
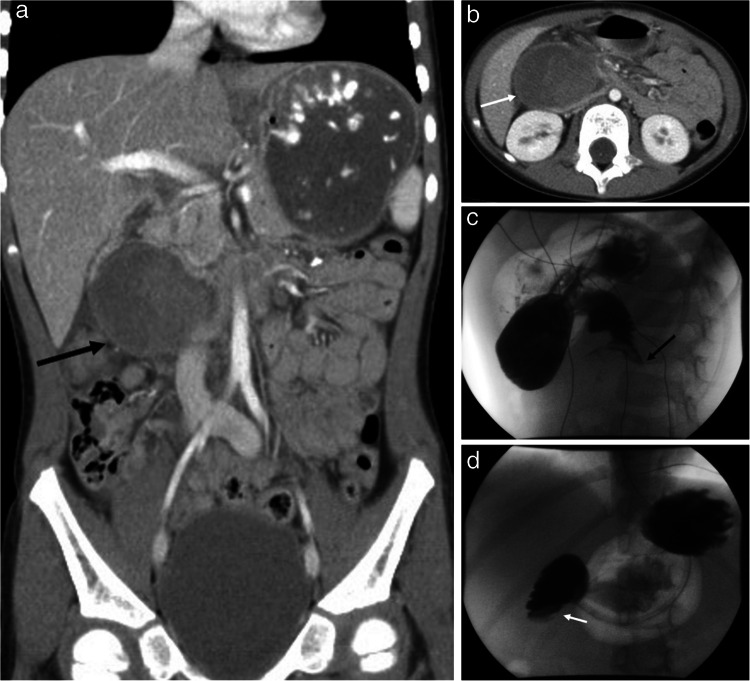
A 4-year-old female without a history of trauma or bleeding and clotting disorders presents with bilious emesis and feeding intolerance. A CT of the abdomen and pelvis with IV contrast and oral contrast in portal venous phase was performed and which elicited concern for physical abuse. An upper GI exam was also performed a few hours after the CT. **a** Coronal CT image of a shows a large heterogeneously hypodense intraluminal lesion at the junction of the second and third duodenal segments compatible with a duodenal hematoma (*black arrow*) with upstream fluid distention of the more proximal duodenum and stomach which is distended with fluid and recently ingested oral contrast. **b** Axial image of the same study shows this large intraluminal duodenal hematoma at the junction of the second and third duodenal segments (*white arrow*). **c** A lateral projection image from the upper GI exam shows this duodenal hematoma (*black arrow*) to be obstructing with no significant passage of contrast into the duodenum distal to this obstructing hematoma. **d** A frontal projection image from the upper GI exam shows no distal transit of contrast beyond the second portion of the duodenum, site of hematoma (*white arrow*)

Duodenal hematomas are more common than duodenal perforations and may present with gastric outlet obstruction and feeding intolerance [5, 10, 59]. Proximal jejunal perforations have also been reported with abusive injury [5, 9]. It is important to distinguish between perforations and hematomas, as perforations require operative repair while hematomas can be managed conservatively with bowel rest, parenteral nutrition, and enteric tube decompression of the stomach. However, it may be difficult to distinguish between a duodenal hematoma and a perforation on imaging. A duodenal perforation should be suspected if CT imaging reveals a retroperitoneal collection of contrast medium, extraluminal gas, or a lack of continuity of the duodenal wall [48, 60]. Clinical signs and symptoms of inflicted duodenal injury can manifest as abdominal pain, abdominal distention, nausea, bilious or nonbilious vomiting, and the inability to tolerate oral feeds. Oral contrast should be administered whenever there is a clinical suspicion for inflicted duodenal injury. Administering 8–10 oz of oral water-soluble contrast 30 min prior to obtaining the CT of the abdomen and pelvis with IV contrast can aid in distinguishing perforations from hematomas in the proximal small bowel [14, 61]. In some cases, an upper GI series may be needed following CT to further distinguish and clarify equivocal CT findings [14, 61] (see Figs. 8, 9, and 10).

### Skeletal injuries

While the CT of the abdomen and pelvis is primarily used to assess intra-abdominal organs, it can occasionally reveal incidental skeletal injuries. Special care should be taken to thoroughly review injuries to the visualized lower ribs, spine, pelvic bones, and upper femurs. Rib fractures in infants carry high specificity for abuse, and incidental skeletal injuries should prompt consideration of child physical abuse in the appropriate clinical context [62, 63].

### Hypoperfusion complex

Hypoperfusion complex describes a constellation of abdominal CT imaging findings that indicate a state of tenuous compensated shock. Thus, while some children may appear clinically and hemodynamically stable, they are in a fragile state with the potential to rapidly deteriorate. Prompt recognition of hypoperfusion complex on imaging is crucial to initiate aggressive resuscitation measures. Children with hypoperfusion complex frequently present with concomitant intra-abdominal and intracranial injury [64]. Hypoperfusion complex is a predictor of poor outcome with a reported mortality of up to 85% in children [64].

**Fig. 11:**
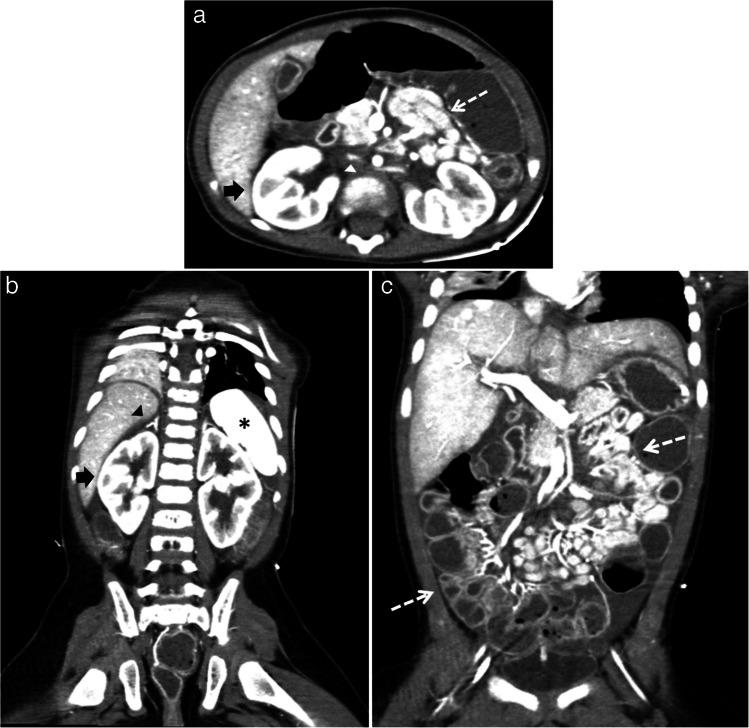
An 11-month-old female found unresponsive after reportedly falling off a couch at home. CT head found significant traumatic brain injury. Further trauma work was obtained with CT of the abdomen and pelvis with IV contrast in the portal venous phase, which demonstrated a constellation of findings consistent with hypoperfusion complex. **a** Axial image shows hyperdense kidneys (*black arrow*), hyperdense bowel (*dashed white arrow*), and intensely enhancing, small caliber IVC (*white arrowhead*). **b** Coronal image shows hyperdense kidneys (*black arrow*), adrenal glands (*black arrowhead*), and spleen (*asterisk*). **c** Coronal image of the same study at a different plane shows fluid-filled and hyperdense bowel wall (*dashed arrow*). Note the intensely enhancing mesenteric vessels, which extend into the serosal margin of bowel loops. In summary, trauma workup discovered multiple injuries including subdural hemorrhages, bilateral retinal hemorrhages, healing rib fractures, liver laceration, and bruising to the face and body, consistent with an inflicted abusive injury mechanism. The patient was declared brain dead and expired within 48 hours of presentation

In states of shock, the sympathetic nervous system becomes activated, redirecting blood from the cutaneous, visceral, and muscular circulatory beds to the kidneys, heart, and brain. This altered perfusion manifests in a constellation of both classic and variable findings that constitute the hypoperfusion complex on the CT of the abdomen and pelvis with IV contrast performed in the portal venous phase. Findings include diffuse dilated, fluid-filled bowel with hyperenhancement of the bowel wall (termed “shock bowel”), intense enhancement of small caliber abdominal aorta and inferior vena cava (IVC), and variable enhancement of solid organs, either increased or decreased from their usual enhancement appearance. Both hyperenhancement of the kidney and complete lack of enhancement (“black kidney” sign) have been reported [65]. Other reported findings are hyperenhancement of the adrenal glands and pancreas, hypoperfusion of the spleen and pancreas, intensely enhancing small caliber superior mesenteric artery, persistent ureteral enhancement, and peritoneal and retroperitoneal fluid [64]. Increased concentration of contrast medium in depleted intravascular volume may explain why some organs display hyperenhancement in this complex (see Fig. 11).

Differentiation of hypoperfusion complex and bowel injury is important to direct appropriate clinical care. Both entities have overlapping imaging findings, but bowel injury will have focal bowel and mesentery changes whereas hypoperfusion complex will have more diffuse bowel, visceral, and vascular findings.

## Patient outcomes

Intra-abdominal injuries are important clinical findings identified in pediatric fatalities from physical abuse. Reported fatality rates in children with inflicted abdominal trauma vary and may be complicated by the presence of concurrent injuries. A study of 1995–2001 data from the National Pediatric Trauma Registry (NPTR) assessed outcomes in 927 hospitalized children under 5 years old with blunt abdominal injury from abusive or accidental mechanisms, excluding motor vehicle collisions (MVC) [8]. In this study, 21.9% of 146 children with abuse and intra-abdominal injuries died compared to 9.7% of the overall population with all-cause mechanisms excluding MVC. Those with intra-abdominal injury and concurrent traumatic brain injury (TBI) had higher death rates than those with abdominal injuries alone. Abuse remained an important risk factor for death even when considering concurrent injuries and adjusting for multiple factors including sex, age, and injury mechanism. Abuse was associated with sixfold the odds of death compared to falls.

More recent studies have demonstrated lower fatality rates for inflicted intra-abdominal injuries than those reported in early work. A study of children hospitalized with abdominal trauma found fatalities to be 9% in children with inflicted intra-abdominal injuries versus 3.4% of children with intra-abdominal injuries that were non-inflicted, or accidental [66]. Another multicenter study of > 2000 children undergoing subspecialty child physical abuse evaluations identified 82 (2.8%) children with intra-abdominal injuries not limited to abuse, of which 9 (11%) died [4]. A recent study of children with intra-abdominal injuries reported mortality rates of 10.3% in cases of abuse compared to 7.1% in cases of accidents, which was not found to be significantly different [13]. In addition, a recent study of children undergoing physical abuse evaluations without history, signs, or symptoms of abdominal trauma found that 1 (6%) of 16 children with occult abdominal trauma died. This child had a grade III hepatic injury in the setting of multiple concurrent injuries, including TBI [23]. These lower mortality rates may reflect variations in the study populations and screening practices. As screening for intra-abdominal injuries in suspected physical abuse has likely become more widespread, it is possible that detection of less severe injuries has increased. While such less severe injuries may be relevant for the diagnosis of abuse, they are less likely to impact the patient’s overall health or survival than severe concomitant injuries, such as intracranial trauma. This possible shift in detection may explain some of the observed changes in reported mortality.

## Conclusion

Physical abuse is an important cause of intra-abdominal injuries, especially in young children. Given the broad spectrum of presenting signs and symptoms, healthcare providers must be aware of the clinical manifestations of intra-abdominal injuries and should familiarize themselves with common radiological imaging modalities. While certain injuries may be more prevalent in cases of physical abuse, none are pathognomonic. Providers should therefore carefully assess intra-abdominal injuries in the context of the patient’s age, developmental stage, history provided, and the overall constellation of identified injuries. When concern for physical abuse arises, close collaboration among radiologists, child abuse pediatricians, and other members of the clinical team can aid in interpreting clinical findings. A multidisciplinary approach can help ensure a thorough evaluation essential for accurate diagnosis and effective management.

## Data Availability

No datasets were generated or analysed during the current study.

## Abbreviations

TBI: Traumatic brain injury
MVC: Motor vehicle collisions
CT: Computed tomography
US: Ultrasound
CEUS: Contrast-enhanced ultrasound
ACR: American College of Radiology
FAST: Focused assessment with sonography for trauma
IVC: Inferior vena cava
AAST: American Association for the Surgery of Trauma
